# Draft Genome Sequence of *Aeromonas caviae* Strain 429865 INP, Isolated from a Mexican Patient

**DOI:** 10.1128/genomeA.01240-15

**Published:** 2015-10-22

**Authors:** Juan Carlos A. Padilla, Patricia Bustos, Graciela Castro-Escarpulli, Alejandro Sánchez-Varela, Ingrid Palma-Martinez, Patricia Arzate-Barbosa, Carlos A. García-Pérez, María de Jesús López-López, Víctor González, Xianwu Guo

**Affiliations:** a Centro de Biotecnología Genómica, Instituto Politécnico Nacional, Reynosa, Tamaulipas, México; b Centro de Ciencias Genómicas, Universidad Nacional Autónoma de México, Cuernavaca, Morelos, México; c Laboratorio de Bacteriología Médico, Escuela Nacional de Ciencias Biológicas, Instituto Politécnico Nacional, Ciudad de México, México; d Departamento de Análisis Clínicos y Estudios Especiales, Instituto Nacional de Pediatría, Ciudad de México, México

## Abstract

*Aeromonas caviae* is an emerging human pathogen. Here, we report the draft genome sequence of *Aeromonas caviae* strain 429865 INP which shows the presence of various putative virulence-related genes.

## GENOME ANNOUNCEMENT

*Aeromonas caviae* is a rod-shaped, Gram-negative, ubiquitous bacterium which has been implicated in a variety of intestinal and extraintestinal illnesses as well as soft-tissue infections in humans. It is also one of the bacteria in the genus *Aeromonas* responsible for the majority (>85%) of human infections and clinical isolates (1) and is related to the most frequenty isolated species in pediatric patients (2, 3). Here, we report the sequenced genome of *A. caviae* strain 429865 INP isolated from an infected 2-year and 4-month-old patient in Mexico.

The genomic DNA of *A. caviae* strain 429865 INP was extracted using the Wizard genomic DNA purification kit A1120 (Promega Corp., Madison, WI, USA) according to the manufacturer’s recommended protocol. The purified genomic DNA was measured using the NanoDrop spectrophotometer and Qubit 2.0 fluorometer (Thermo, Fisher Scientific, Waltham, MA) to verify quality and purity. The whole genome sequencing was performed using the Illumina MiSeq platform (Illumina, Inc., San Diego, CA, USA) according to the standard operation based on a paired-end library and a mate-pair library of 5-Kb fragments. The resulting sequence reads were inspected for data quality using FASTQC v0.11.3 (Babraham Institute, Cambridge, United Kingdom) and were then filtered using the DynamicTrim tool from SolexaQA v3.1.3 (4) to remove and dynamically trim poor reads. The reads were then assembled *de novo* using SPAdes v3.5.0 (5) which resulted in 204 contigs. The resulting contigs were aligned with the original reads using Consed v29.0 (6) and through this method were manually joined together *in silico*. The resulting draft genome sequence of *A. caviae* strain 429865 INP is 4,700,593 bp in length and consists of 4 contigs with an average coverage of 936.05-fold. The mean contig read length is 1,175,148.25 bp and the maximum contig read length is 2,239,764 bp. The overall G+C content of the assembled genome is 61.0%.

The NCBI Prokaryotic Genome Annotation Pipeline was used for annotation of the resulting draft genome sequence of *A. caviae* strain 429865 INP. This resulted in the identification of 4,014 putative coding sequences (CDS), 10 16S-23S-5S operons, and 114 tRNAs. Various putative genes that could be involved in the pathogenicity mechanism of the bacteria were identified, including the cytotonic enterotoxin (*alt*), extracellular lipase (*lip*), proteins related to motility such as polar flagellar proteins (*flaA*, *flaB*) and lateral flagellar proteins (*lafA1*, *lafA2*), a type II secretion system, genes associated with drug resistance such as bicyclomycin resistance protein (AHA_2550) and fosmidomycin resistance protein (AHA_0056), and various multidrug resistance efflux pumps. This genome provides a useful guide to study the pathogenesis of this organism.

### Nucleotide sequence accession numbers.

The draft genome sequence of *Aeromonas caviae* strain 429865 INP has been deposited at DDBJ/EMBL/GenBank under the accession number LIIX00000000. The version described in this paper is version LIIX01000000.
